# Promotion of the mind through exercise (PROMoTE): a proof-of-concept randomized controlled trial of aerobic exercise training in older adults with vascular cognitive impairment

**DOI:** 10.1186/1471-2377-10-14

**Published:** 2010-02-17

**Authors:** Teresa Liu-Ambrose, Janice J Eng, Lara A Boyd, Claudia Jacova, Jennifer C Davis, Stirling Bryan, Philip Lee, Penny Brasher, Ging-Yuek R Hsiung

**Affiliations:** 1 Department of Physical Therapy, University of British Columbia, Vancouver, Canada; 2 Centre for Hip Health and Mobility, Vancouver Coastal Research Institute, Vancouver, Canada; 3 Division of Neurology, University of British Columbia, Vancouver, Canada; 4 Experimental Medicine, University of British Columbia, Vancouver, Canada; 5 Centre for Clinical Epidemiology and Evaluation, Vancouver Coastal Research Institute, Vancouver, Canada; 6 Division of Geriatric Medicine, University of British Columbia, Vancouver, Canada

## Abstract

**Background:**

Sub-cortical vascular ischaemia is the second most common etiology contributing to cognitive impairment in older adults, and is frequently under-diagnosed and under-treated. Although evidence is mounting that exercise has benefits for cognitive function among seniors, very few randomized controlled trials of exercise have been conducted in populations at high-risk for progression to dementia. Aerobic-based exercise training may be of specific benefit in delaying the progression of cognitive decline among seniors with vascular cognitive impairment by reducing key vascular risk factors associated with metabolic syndrome. Thus, we aim to carry out a proof-of-concept single-blinded randomized controlled trial primarily designed to provide preliminary evidence of efficacy aerobic-based exercise training program on cognitive and everyday function among older adults with mild sub-cortical ischaemic vascular cognitive impairment.

**Methods/Design:**

A proof-of-concept single-blinded randomized trial comparing a six-month, thrice-weekly, aerobic-based exercise training group with usual care on cognitive and everyday function. Seventy older adults who meet the diagnostic criteria for sub-cortical ischaemic vascular cognitive impairment as outlined by Erkinjuntti and colleagues will be recruited from a memory clinic of a metropolitan hospital. The aerobic-based exercise training will last for 6 months. Participants will be followed for an additional six months after the cessation of exercise training.

**Discussion:**

This research will be an important first step in quantifying the effect of an exercise intervention on cognitive and daily function among seniors with sub-cortical ischaemic vascular cognitive impairment, a recognized risk state for progression to dementia. Exercise has the potential to be an effective, inexpensive, and accessible intervention strategy with minimal adverse effects. Reducing the rate of cognitive decline among seniors with sub-cortical ischaemic vascular cognitive impairment could preserve independent functioning and health related quality of life in this population. This, in turn, could lead to reduced health care resource utilization costs and avoidance of early institutional care.

**Trial Registration:**

ClinicalTrials.gov Protocol Registration System: NCT01027858.

## Background

Cerebrovascular disease is the second most common etiology contributing to dementia in older adults [1-4] and may be the most under-diagnosed and yet most treatable form of cognitive dysfunction in older adults [5]. Vascular cognitive impairment is defined as the loss of cognitive function resulting from ischaemic, ischaemic-hypoxic, or hemorrhagic brain lesions as a result of cerebrovascular disease and cardiovascular pathologic changes. As vascular cognitive impairment is predominantly a sub-cortical frontal form of cognitive disorder with prominent executive dysfunction [6], it directly impairs everyday function [7], such as managing finances, transportation, or the telephone. Taken together, vascular cognitive impairment has the potential to severely impact the ability to function autonomously within society [8].

A number of epidemiological studies suggest that modification of vascular risk factors, such as hypertension, diabetes mellitus, and hypercholesterolemia may be helpful in slowing down the progression of vascular cognitive impairment [9-12]. Hence, one promising approach to delay the progression of vascular cognitive impairment is aerobic-based exercise training. Critically, evidence is mounting that exercise has benefits for cognitive function among seniors. Aerobic-based exercise training may be of specific benefit in slowing the progression of cognitive decline among seniors with vascular cognitive impairment by reducing key vascular risk factors associated with metabolic syndrome. Aerobic exercise may also act specifically on disease pathophysiology [13], for example, by improving capillarization or modulating brain neurotrophic factors [14-16], and may even decrease brain amyloid load in the brain [17]. Finally, aerobic-based exercise training as an intervention strategy for individuals with vascular cognitive impairment is attractive as it could be delivered at a population-level.

The growing consensus is that small vessel diseases have an important role in vascular cognitive impairment [18,19]. Small vessel disease often presents as undetected "covert" strokes in the sub-cortical white matter. Compared to other forms of vascular cognitive impairment, the sub-cortical ischaemic vascular disease sub-type is a more homogenous form of the disease with a more predictable outcome [20]. While mild **S**ub-cortical **I**schaemic **V**ascular **C**ognitive **I**mpairment (SIVCI) describes those individuals whose symptoms are not associated with substantial functional impairment [18], persons with mild SIVCI have a high risk of progression to dementia [18,21]. Seniors with mild SIVCI are also at risk for executive dysfunction [18], and subsequently at risk for decline in everyday function (i.e., ability to perform instrumental activities of daily living [ADLs]) [7]. Instrumental ADLs refer to capacities that are required for autonomous function within society [8]; the ability to perform instrumental ADLs is a key aspect of functional independence and health related quality of life [8]. Thus, persons with mild SIVCI represent an ideal target population for intervention strategies, as preservation of their cognitive and functional status will likely maintain and prolong their ability to live independently and with quality. To our knowledge, no randomized controlled trial has assessed the effect of aerobic-based exercise training on cognitive and everyday function among seniors with mild SIVCI. Thus, we aim to carry out a proof-of-concept single-blinded randomized controlled trial primarily designed to provide preliminary evidence of efficacy of a six-month, thrice-weekly, aerobic-based exercise training program on cognitive and everyday function among older adults with mild SIVCI. In addition, this proof-of-concept study will aim to explore the effect of aerobic-based exercise training on: 1) serum glucose, hemoglobin A1c, and lipids; 2) inflammatory biomarkers; 3) physical function; 4) health related quality of life; and 5) health resource utilization. Finally, this study will demonstrate the feasibility of delivering the intervention in this population, determine the recruitment rate, determine the rate of withdrawal, and provide estimates of the variances, co-variances and effect sizes of the proposed outcome measures to inform the sample size for a larger definitive trial.

## Methods/Design

### Design Outline

**Figure 1:**
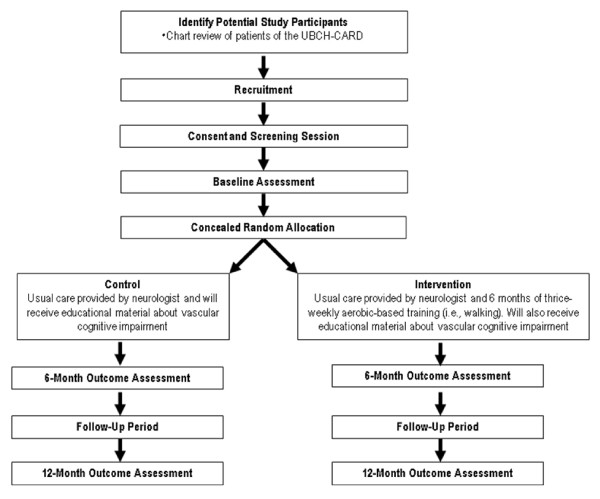
**Overview of the flow of participants through the Promotion of the Mind Through Exercise (PROMoTE) Trial**.

We will conduct a six-month proof-of-concept single-blinded randomized trial and follow our study cohort for an additional six months of follow-up (see Figure 1).

### Recruitment

We will recruit seniors with mild SIVCI through the University of British Columbia Hospital Clinic for Alzheimer Disease and Related Disorders (UBCH-CARD). All individuals who receive care at the UBCH-CARD have the option to sign a consent form providing access to their records for research purposes and indicating their willingness to be approached for research studies. One trained research assistant will review the charts of patients at the UBCH-CARD who have expressed interests to participate in research studies. Clinicians at the UBCH-CARD will also be informed of this study and they will assist in recruitment by flagging the charts of their current patients who may qualify for this study to our research assistants. Those who appear eligible based on detailed chart review will be mailed an information package regarding the study, including the consent form.

For those who are interested in participating, a one hour consent and screening session will be arranged. Once informed consent is obtained, we will perform the screening tests. Those who remain eligible after the screening session and who later provide written recommendation from their physician indicating their appropriateness to participate in an aerobic-based exercise training program will proceed to baseline assessments.

### Eligibility

We will recruit individuals who fulfill the diagnostic criteria for SIVCI as outlined by Erkinjuntti and colleagues [22], which requires the presence of both cognitive syndrome (as defined in Section A below) and small vessel ischaemic disease (as defined in Section B below).

A. Cognitive Syndrome defined as:

1. *Dysexecutive Syndrome: *Some impairment in goal formulation, initiation, planning, organizing, sequencing, executing, set-shifting and maintenance, or abstracting.

2. *Memory Deficit: *Some impairment in recall in the presence of relatively intact recognition and benefit from cueing.

3. *Progression: *Deterioration of A1 and A2 from a previous higher level of functioning that are not per se interfering with complex occupational and social activities.

B. Small Vessel Ischaemic Disease defined as:

1. *Evidence of relevant cerebrovascular disease by brain imaging *(in the last 12 months) defined as the presence of both:

i. Periventricular and deep white matter lesions: Patchy areas of low attenuation (intermediate density between that of normal white matter and that of intraventricular cerebrospinal fluid) or diffuse symmetrical areas of low attenuation with ill defined margins extending to the centrum semiovale, plus at least one lacunar infarct (correlating to the white matter grading scale greater than 3 from the Cardiovascular Health Study) [23,24]; and

ii. Absence of cortical and/or cortico-sub-cortical non-lacunar territorial infarcts and watershed infarcts, haemorrhages indicating large vessel disease, signs of normal pressure hydrocephalus, or other specific causes of white matter lesions (e.g., multiple sclerosis, leukodystrophies, sarcoidosis, brain irradiation, etc).

2. *Presence or a history of neurological signs *as evidence for cerebrovascular disease such as lower facial weakness, Babinski sign, sensory deficit, dysarthria, gait disorder, or extrapyramidal signs consistent with sub-cortical brain lesion(s).

In addition, individuals must meet the following inclusion criteria: 1) Montreal Cognitive Assessment (MoCA) [25] score less than 26 at screening; 2) Mini-Mental State Examination (MMSE) [26] score of ≥ 20 at screening; 3) Community-dwelling; 4) Live in Metro Vancouver; 5) Have a caregiver, family member, or friend who interacts with him/her on a weekly basis; 6) Able to comply with scheduled visits, treatment plan, and other trial procedures; 7) Must be able to read, write, and speak English in which psychometric tests are provided with acceptable visual and auditory acuity; 8) Stable on a fixed dose of cognitive medications (e.g., donepezil, galantamine, rivastigmine, memantine, etc.) that is not expected to change during the 12-month study period, or, if they are not on any of these medications, they are not expected to start them during the 12-month study period; 9) Provide a personally signed and dated informed consent document indicating that the individual (or a legally acceptable representative) has been informed of all pertinent aspects of the trial. In addition, an assent form will be provided at baseline and again at regular intervals; 10) Able to walk independently; and 11) Must be in sufficient health to participate in the study's aerobic-based exercise training program. This will be based on medical history, vital signs, physical examination by study physicians, and written recommendation by family physician indicating one's appropriateness to participate in an aerobic-based exercise training program.

The exclusion criteria are: 1) Absence of relevant small vessel ischaemic lesions on an existing brain computed tomography (CT) or magnetic resonance imaging (MRI); 2) Diagnosed with another type of neurodegenerative (e.g. AD) or neurological condition (e.g., multiple sclerosis, Parkinson's disease, etc.) that affects cognition and mobility; 3) At high risk for cardiac complications during exercise and/or unable to self-regulate activity or to understand recommended activity level (i.e., Class C of the American Heart Risk Stratification Criteria); 4) Have clinically significant peripheral neuropathy or severe musculoskeletal or joint disease that impairs mobility; 5) Taking medications that may negatively affect cognitive function, such as anticholinergics, including agents with pronounced anticholinergic properties (e.g., amitriptyline), major tranquilizers (typical and atypical antipsychotics), and anticonvulsants (e.g., gabapentin, valproic acid, etc.); or 6) Planning to participate, or already enrolled in, a clinical drug trial concurrent to this study.

Ethical approval has been obtained from the Vancouver Coastal Health Research Institute (V07-01160) and the University of British Columbia's Clinical Research Ethics Board (H07-01160).

### Sample Size

Prior to launching a definitive randomized controlled trial, it is essential that the feasibility of conducting such a trial be demonstrated. A sample for a definitive study with multiple end-points and outcome variables will require several hundred participants and warrant a multi-site study. Given that no study has examined the effect of exercise on cognitive function in SIVCI, we have selected a sample size of 30 participants per group. Studies have suggested a standard deviation of change of 6 to 7 [27-29] in Alzheimer Disease Assessment Scale Cognitive (ADAS-Cog) scores among individuals with vascular cognitive impairment. We highlight that a recent exercise trial among seniors at risk for AD with ADAS-Cog as the primary outcome measure demonstrated an effect size of 0.60 [30]. Thus, assuming an alpha of 0.05, 30 participants per group will provide a power of 0.75 to 0.80. We estimate a drop-out rate of 10% during a 12-month period. Hence, we are aiming to recruit 35 participants per group (i.e., 70 participants in total) which will accommodate a conservative 15% drop-out rate.

### Measurements

Baseline measurements will be obtained prior to randomization. There will be three measurement sessions: baseline, 6 months, and 12 months (Figure 1). Outcomes will be assessed by trained assessors blinded to group allocation.

#### Screening Session

For the screening session, we will administer the Physical Activity Readiness Questionnaire (PAR-Q) [31], a screening measure of physical readiness for exercise. Global cognitive function will be assessed using the MMSE [26] and the MoCA [25].

#### Descriptors

Using a wall mounted stadiometer, standing height will be measured as stretch stature to the 0.1 cm per standard protocol. Weight will be measured twice to the 0.1 kg on a calibrated digital scale. General health and socioeconomic status will be ascertained by a questionnaire. Participants will undergo a clinical assessment with neurologist and study physicians (G-YRH and PL) at baseline to confirm current health status and eligibility for study, including clinical impressions of overall cognitive and functional status. The Neuropsychiatric Inventory (NPI), an informant-rated instrument, will be used to evaluate behavioural and neuropsychiatric symptoms [32].

Physical activity, not including the study-assigned exercise classes, will be determined by the valid and reliable Physical Activities Scale for the Elderly (PASE) questionnaire [33,34]. Designed for those aged 65 years and older, participants use a 12-item scale to self-report the average number of hours per day spent participating in leisure, household, and occupational physical activities over the previous seven-day period. Accounting for extracurricular physical activities throughout the randomized trial is essential to ascertain the specific effects of the delivered interventions on cognition and function.

#### Primary Outcomes

##### Cognitive Function 

We will assess cognitive function using the cognitive section of the Alzheimer Disease Assessment Scale (ADAS-Cog) [35]. The scale consists of 11 brief cognitive tests assessing memory, language, and praxis. Scores range from 0 to 70, with higher scores indicating greater severity of cognitive impairment. The ADAS-Cog has been a significant outcome measure in numerous trials with AD [27,36,37] but also with vascular dementia [29,38]. The ADAS-Cog has marked advantages as an outcome measure, based on its substantial data confirming both reliability and validity and its use in measuring longitudinal change together with sensitivity to treatment effects [39].

##### Global Executive Function 

Because executive dysfunction is common among those with SIVCI [6], we will use the Executive Interview (EXIT-25) [40] to assess global executive function. The EXIT 25 provides a standardized clinical assessment of executive functions. It contains 25 items designed to elicit signs of frontal system pathology. Performance on the EXIT25 correlates well with standard neuropsychological tests of executive functions. The EXIT25 scores range from 0 to 50, with high scores indicating impaired global executive function. A cut point of 15 out of 50 is recommended [40].

##### Everyday Function

Because executive dysfunction is associated with impaired everyday function [7], we will use the ADCS-ADL to assess the ability to perform everyday activities of daily living [41]. The ADCS-ADL is a 23-item informant-rated questionnaire that measures, in a range of 0 to 78, an individual's performance of activities of daily living.

#### Secondary Outcomes

##### Blood Biomarkers 

Serum glucose, Hgb A1c, and lipid level will be measured by conventional methods. Serum HSC, CRP, and IL-6 will be determined by standard ELISA methods [42].

##### Physical Function 

Physical function will be assessed using a three-instrument performance battery that includes:

1) Six-Minute Walk 

This is a walking test of physical status to assess general cardiovascular capacity in seniors [43]. The total distance walked in meters in six minutes is recorded in meters.

2) Balance and Mobility 

General balance and mobility will be assessed with the National Institute on Aging (NIA) Balance Scale [44]. For this Scale, participants are assessed on performances of standing balance, walking, and sit-to-stand. Each component is rated out of four points, for a maximum of 12 points. Poor performance on this scale predicts subsequent disability [44].

3) Physiological Falls Risk 

We will use the Physiological Profile Assessment (PPA)^© ^[45] (Prince of Wales Medical Research Institute, Randwick, Sydney, NSW, Australia) to assess for physiological falls risk. The PPA is a valid and reliable tool for assessing fall risk in older people. Based on the performance of five physiological domains (postural sway, hand reaction time, quadriceps strength, proprioception, and edge contrast sensitivity), the PPA computes a fall risk score for each individual and this measure has a 75% predictive accuracy for falls in older people [45]. A PPA z-score of 0-1 indicates mild risk, 1-2 indicates moderate risk, 2-3 indicates high risk, and 3 and above indicates marked risk [46].

4) Quality of Life 

We will evaluate health related quality of life using the EuroQol 5D (EQ-5D) -- a preference-based generic utility instrument that provides weightings for quality adjusted life year (QALYs). QALYs are defined as the benefit of a health intervention in terms of time in a series of quality-weighted health states, in which the quality weights reflect the desirability of living in the state, typically from "perfect" health (weighted 1.0) to dead (weighted 0.0) [47]. QALYs are calculated based on the quality of life of a patient (measured using health utilities) in a given health state and the time spent in that health state. The EQ-5D captures 243 unique health states based on the following domains: 1) mobility; 2) self-care; 3) usual activities; 4) pain and 5) anxiety or depression. We will calculate QALYs using the health state utility values from the EQ-5D to determine if there is a statistically significant difference in the incremental cost per QALY change for participants receiving the aerobic-based training compared with those who are not.

5) Health Resource Utilization 

The health resource utilization questionnaire asks participants to report the following visits over a specified time period: 1) health care professionals; 2) admissions or visits to hospital; and 3) laboratory work. The health resource utilization questionnaire has been previously described and supported in previous studies [48]. We will estimate total health care related costs over the 12 months from a Canadian health care system perspective. Participants will be instructed to specify total health care expenditure and report the reason for each item. Additionally, participants will be instructed to report health care expenditure due to any adverse events associated with the aerobic-based training program; this is not anticipated to be a major cost driver. On a per participant basis, costs will be assigned to health care resource utilization using a fully allocated hospital cost model (for in-patient costs) and the British Columbia provincial guide to medical fees (for outpatient costs). Our base case analysis will consider the costs of all health care resource use and our sensitivity analyses will include only intervention related health care resource costs and a complete case analysis.

### Treatment Allocation

#### Randomization

Participants will be randomly assigned (1:1) to either the aerobic-based exercise training (AT) group or the usual care (CON) group. The randomization sequence will be generated by a central, web-based randomization service http://www.randomization.net; permuted blocks of varying size will be employed to ensure balance over time. After baseline assessment, research personnel not involved in measurement or intervention will access the web-based randomization service to determine the group allocation.

#### Allocation Concealment

Recruitment and enrolment of participants will be managed by the research coordinator who will screen for study eligibility, obtain informed consent, and conduct baseline assessment. Following completion of baseline assessment, the research coordinator will access the web-based randomization service and the participant will be assigned a participant number and allocated to the intervention or the control group. Research personnel performing the outcome assessment and data analysis will be blinded to group allocation but it is not possible to blind participants and personnel delivering interventions to group allocation.

### Experimental Groups

#### Aerobic-Based Training (AT) Group

All AT group classes will be led by instructors certified to instruct seniors. Each 60-minute class will include a 10 minute warm-up, a target of 40 minutes of walking, and a 10-minute cool down. Class attendance will be recorded for each participant by the instructors throughout the six-month intervention period.

Over the six month intervention period, we will use three complimentary techniques to monitor and progress exercise intensity of the AT program. Each participant will:

1) Wear a heart rate monitor and will be asked to work initially at approximately 40% of his/her age specific target heart rate (i.e., heart rate reserve; HRR) and gradually progress to reach the target of 60% of HRR. Once the target of 60% of HRR is achieved, it will be sustained by the participant for the remainder of the intervention period;

2) Subjectively monitor the intensity of each workout using the Borg's Rating of Perceived Exertion (RPE) [49]. Participants will be gradually progressed to a target RPE of 14 to 15; and

3) Use the simple "talk" test [50,51]. Participants will be asked to initially walk at a pace where they can converse comfortably without effort and gradually progress to a pace where conversation requires a bit of effort.

In addition to the formal group classes, all individuals in the AT group will be given a pedometer to serve as both an incentive and reminder to partake in walking on a daily basis. Participants will be asked to record the number of steps each day. All pedometers will be returned to the study coordinator at the 6 month assessment session.

#### Usual Care (CON) Group

Participants in the CON group will receive educational material about vascular cognitive impairment and about stress management, healthful diet, and smoking. However, this group will not receive specific information regarding physical activity. Participants in the AT group will also be offered these educational materials.

### Adverse Events Monitoring

Dr. Philip Lee, in addition to a physician and statistician external to the research team, will review all adverse events reported in the study on a monthly basis. They will inform us to stop the study if the adverse events data are of sufficient concern.

### Statistical Analyses

As a primary objective of this study is to provide preliminary evidence of **efficacy **we will compare participants of the AT group who are compliant with the intervention (defined as attending 60% of the total exercise sessions) to the CON group rather than using an intention-to-treat analysis as would be appropriate in a pivotal clinical trial. In addition, no adjustment for multiple endpoints will be made since in a proof-of-concept study a Type II error is of more concern than a Type I error [52]. For each of the three primary endpoints (i.e., ADAS-Cog, EXIT-25, ADCS-ADL), the change from baseline to six months and 12 months will be assessed using an analysis of covariance model incorporating the baseline measurement. Observing a statistically significant difference on **any **of the three primary outcomes will be considered preliminary evidence of efficacy.

We will also report variances, co-variances, and effect sizes, as well as sampling feasibility (i.e., ease of recruitment, recruitment rate, withdrawal rate). This information will inform sampling for future trials.

### Economic Analysis

Our economic evaluation will explore incremental costs and health benefits comparing the aerobic-based training intervention with usual care using both cost effectiveness and cost utility analyses. A cost utility analysis is a specific type of cost effectiveness analysis where health benefits are measured using QALYs. The outcome of our cost effectiveness analysis is the incremental cost effectiveness ratio (ICER). By definition, an ICER is the difference between the mean costs of providing the competing interventions divided by the difference in effectiveness, where ICER = Δ Cost/Δ Effect [53]. We will determine the incremental cost of the aerobic-based training intervention per person experiencing a clinically significant improvement in cognitive performance relative to usual care. Further, for our cost utility analysis, we will estimate the incremental cost per quality adjusted life year gained (QALY). We will use a combination of imputation and bootstrapping to quantify uncertainty due to missing values and the finite study sample size^60 61^.

### Time Frame

Recruitment will commence in December 2009. Final follow-up assessment is expected to conclude in February 2012.

## Discussion

Although exercise therapy holds promise for delaying the onset and slowing down the progression of both cognitive and functional decline, very few randomized controlled trials of exercise have been conducted in populations at high-risk for dementia [30]. This research will be an important first step in quantifying the effect of an exercise intervention on cognitive and daily function among seniors with SIVCI, as vascular cognitive impairment is the second most common cause of dementia in older adults [1-4]. Exercise has the potential to be an effective, inexpensive, and accessible intervention strategy with minimal adverse effects. Retarding the rate of cognitive decline among seniors with SIVCI could preserve independent functioning and quality of life in this population. This, in turn, could lead to reduced health care utilization costs and avoidance of early institutional care. Delaying the onset or retarding the progression of dementia by only one year would reduce the number of clinical cases of dementia by 9.2 million by 2050 in the US alone [54].

Our interdisciplinary research team will use a multi-pronged approach to explore the utility of aerobic-based exercise training among seniors with mild SIVCI. The impact of our proposed work may be significant for seniors with mild SIVCI.

## Competing interests

The authors declare that they have no competing interests.

## Authors' contributions

TLA, JJE, LAB, G-YRH, CJ, and JB wrote the grant application that was jointly funded by the Canadian Stroke Network and the Heart and Stroke Foundation of Canada in 2009. JCD, PhD candidate is affiliated with the Promotion of the Mind Through Exercise (PROMoTE) Trial. The grant application formed the bases for the manuscript, which was jointly drafted by TLA and JCD with all other authors contributing to its critical review and approving the final draft.

## Pre-publication history

The pre-publication history for this paper can be accessed here:

http://www.biomedcentral.com/1471-2377/10/14/prepub
